# Arterial stiffness and atrial fibrillation: A review

**DOI:** 10.1016/j.clinsp.2022.100014

**Published:** 2022-03-03

**Authors:** João Gabriel Batista Lage, Alexandre Lemos Bortolotto, Mauricio Ibrahim Scanavacca, Luiz Aparecido Bortolotto, Francisco Carlos da Costa Darrieux

**Affiliations:** Instituto do Coração (InCor), Hospital das Clínicas, Faculdade de Medicina, Universidade de São Paulo (HCFMUSP), São Paulo, SP, Brazil

**Keywords:** Atrial fibrillation, Arterial stiffness, Atrial arrhythmia

## Abstract

Arterial stiffness has been investigated as part of the physiopathology of arterial hypertension since the 1970s. Its role in increasing the “pulsatile load” imposed over the Left Ventricle (LV) has been intensely studied recently and has helped in understanding the mechanisms of Atrial Fibrillation (AF) in hypertensive patients. This paper aims to review the main evidence on this issue and establish possible mechanisms involved in the development of AF in patients with arterial stiffness. A PubMed search was performed, and selected articles were searched for references focusing on this topic. In the long term, lower blood pressure levels allow for arterial wall remodeling, leading to a lower stiffness index. To this day, however, there are no available treatments that directly promote the lowering of arterial wall stiffness. Most classes of anti-hypertensive drugs ‒ with stronger evidence for beta-blockers and diuretics ‒ could be effective in reducing arterial stiffness. There is strong evidence demonstrating an association between arterial stiffness and AF. New studies focusing on arterial stiffness and pre-fibrillatory stages would strengthen this causality relation.

## Highlights

• The increase of arterial stiffness at younger ages has been considered a strong predictor of cardiovascular outcomes.
• Elevated “pulsatile load”, which translates into elevated arterial stiffness, would lead to a cascade of phenomena **(left atrium distension, neurohormonal activation and inflammatory response)**, that in their final common path would lead to the development of atrial fibrillation.
• A genetic predisposition for a higher arterial stiffness index was unidirectionally associated with an increase in incidence risk and prevalence of atrial fibrillation. **These results are consistent with a causality association between arterial stiffness and atrial fibrillation.**
• A knowledge gap to be clarified is whether a reduction in arterial stiffness could lead to a decrease in atrial arrhythmia burden, from frequent atrial ectopic beats to atrial fibrillation. **Another knowledge gap is the possible rule of arterial stiffness in the so-called “pre-fibrillatory stage”.**

## Introduction

Atrial Fibrillation (AF) is the most frequent sustained arrhythmia in clinical practice. Although its prevalence varies regionally [1,2], the estimated global burden of AF is around 1‒2% of the population of all ages [3,4]. Discrepancies appear to be explained by differences among study designs but also by racial differences and cultural variations [5].

Among developing countries, AF affects around 0.5% of the population, while its prevalence is estimated to be 3% in the USA, 1.6% in Asia, 4% in Australia and New Zealand, and in Europe, it has been reported to range between 1.9% and 3.3% of the population, depending on the country [1,3,6, 7, 8]. Hypertension and AF share an intimate relationship, since elevated Blood Pressure (BP) is an established risk factor, not only for newly-diagnosed AF, but also for its maintenance and progression 9, 10, 11, and that may be explained, for the most part, by the increase in arterial stiffness.

The role of arterial stiffness in the pathophysiology of hypertension became a focus for research and investigation in the 1970s in France 12, 13, 14, 15. At that time, a series of biochemical and hormonal factors, such as those involved in the renin-angiotensin-aldosterone system causing hypertension were understood, with very little exploration of the mechanisms related to arterial stiffness. The identification of arterial stiffness as a risk factor was an important milestone in the understanding of hypertension, as well as in the development of treatment strategies.

The aim of this paper is to review the role of arterial stiffness in AF development as one independent factor. The present study's objective was to explore the effects of arterial stiffness in left atrium physiology, providing a basis for the understanding of atrial arrhythmias related to hypertension, especially AF.

## Methods

The authors performed a non-systematic comprehensive review of arterial stiffness in patients with AF. A literature search was conducted using the databases PubMed, applying the search terms “Arterial Stiffness and Atrial Arrhythmia” and “Arterial Stiffness and Atrial Fibrillation.” Additionally, reference lists from the available articles were further checked. The studies selected were, for the most part, clinical studies with an observational design. At the same time, articles that were not accessible or only available as an abstract or in a language other than German, English, French, or Spanish were excluded.

### Arterial stiffness assessment

Arterial stiffness is estimated from the aortic or Carotid-Femoral Pulse Wave Velocity (CFPWV), considered the gold-standard method. It is recognized as an independent predictor for future cardiovascular events and all-cause mortality [16,17]. A 1 m/s increase in aortic Pulse Wave Velocity (PWV) leads to 14% and 15% increases in cardiovascular events and mortality, respectively, after adjusting for age, gender, and other risk factors [18], Since aortic PWV is a superior predictor of events to traditional cardiovascular risk factors, it has been proposed that it should be considered an intermediate cardiovascular outcome, not only a regular risk factor [18].

As patients age (the most commonly known risk factor for arterial stiffness), there is a loss of elasticity in the arteries, caused by the substitution of elastin fibers by collagen, which is less elastic, and these changes are associated with the reorganization of cell elements within the vascular wall. The result is a blood vessel less capable of expanding itself to transmit the wave created in the systole during the opening of the aortic valve [18].

**Fig. 1:**
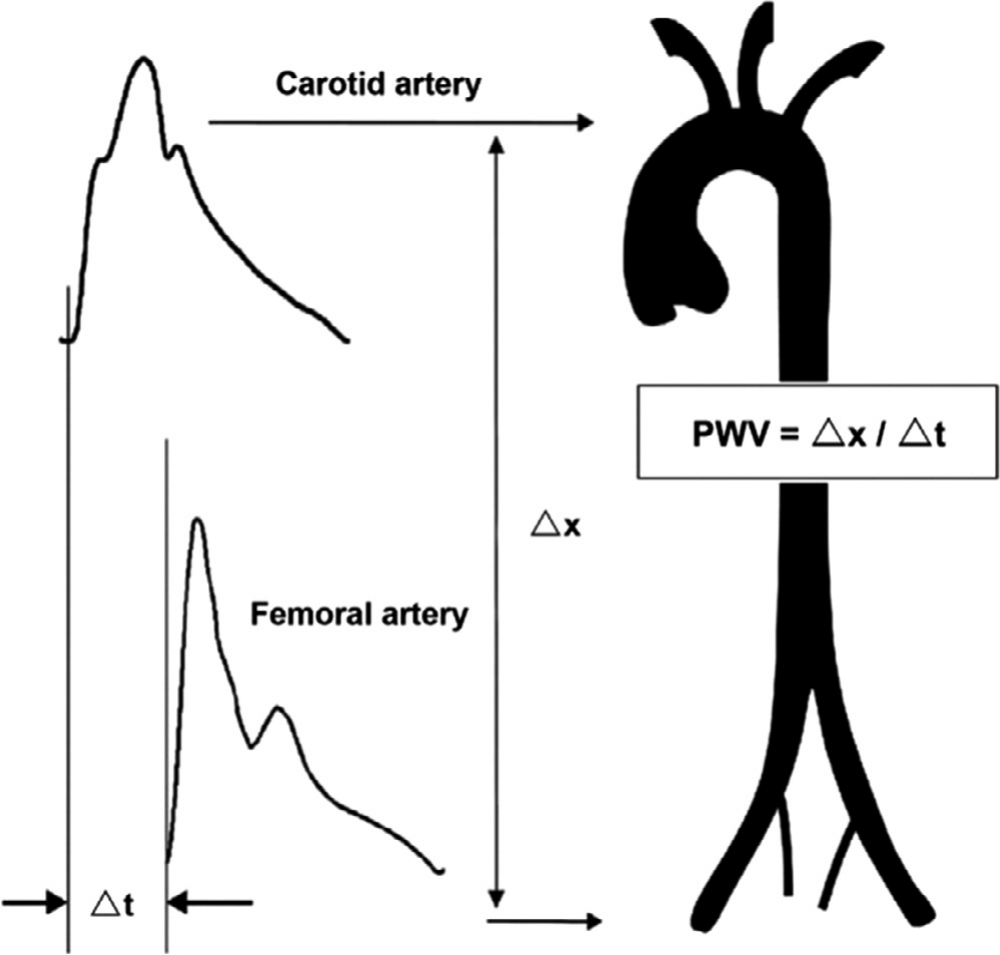
Adapted from Mendes-Pinto et al [19].

PWV measurement is performed by the simultaneous positioning of two mechanographic sensors over the carotid and femoral arteries, located at a known distance. These sensors contain membranes that are successively deformed by the pulse wave shock, and this deformation is initially transformed into an electric signal which is then transmitted to a computer-based calculation program, as shown in Fig. 1.

Table 1 summarizes the main methods for assessing arterial stiffness.

**Table 1:** Methods to assess arterial stiffness.

Carotid-Femoral Pulse Wave Velocity (cfPWV)	Gold-standard method.
Brachial-Ankle Pulse Wave Velocity (baPWV)	baPWV was calculated as: Distance (meters)/Δt (seconds).
Augmentation Index (AI)	Assessed by Applanation Tonometry.The augmentation pressure (AG) is the measure of contribution that the wave reflection makes to the systolic arterial pressure, and it is obtained by measuring the reflected wave coming from the peripheral to the central arteries.
Cardio-Ankle Vascular Index (CAVI)	CAVI is an index obtained by recording the distance from the level of the aortic valve (i.e., brachial level) to the measuring point (i.e., the ankle) and the time delay between the closing of the aortic valve to the detected change in arterial pressure wave at the set point. Information for CAVI computation, including PWV, systolic and diastolic blood pressure as well as arterial pulse waveforms, can then be acquired through the electrocardiogram, cardiac phonogram, and the pressure cuffs on the testing subject at the reference points.Thought to be or claimed to be blood pressure independent.
QKDh	The QKd interval is the time (measured in milliseconds) between the onset of depolarization on electrocardiography (Q) and detection of the last Korotkoff sound (K) at the brachial artery during cuff deflation, corresponding to the diastolic blood pressure (d). This interval is inversely correlated with pulse wave velocity.
β Index	Stiffness Index Beta is derived from PWV, PP, SBP, DBP and viscosity and was calculated by an equation.Thought to be or claimed to be blood pressure independent
Ambulatory Arterial Stiffness Index (AASI)	The Ambulatory Arterial Stiffness Index (AASI) is an indirect arterial stiffness index, which can be simply calculated from 24-h Ambulatory Blood Pressure Monitoring (ABPM).

### Arterial stiffness: epidemiology

Early vascular aging, that is, the increase of arterial stiffness at younger ages, is a strong predictor of cardiovascular outcomes.

Collectively, most studies with ethnic analysis on arterial stiffness demonstrate that populations with African (and many times Hispanic) ancestry have higher arterial stiffness than Caucasian populations, and this has been seen in individuals as young as 6 and as old as 70 years old. Elevated blood pressure frequently accompanies arterial stiffness in Black populations [20,21].

Higher arterial stiffness in black individuals has also been seen in healthy, normotensive people, a phenomenon indicated in studies with normotensive children [20,23,24] and young adults [22]. When analyzing arterial stiffness and aging, it becomes clear that early vascular aging in the black population increases cardiovascular risk in the long term [25,26].

In spite of this, studies reveal only modest evidence of genetic influence over arterial stiffness, with no differences between ethnicities 27, 28, 29. There is also little evidence for maternal risk factors in predicting higher arterial stiffness in children. In contrast, psychosocial factors including racism, lower socioeconomic status, and inadequate health behaviors (such as obesity and sedentary lifestyle) have a significant impact on arterial stiffness 30, 31, 32 and may be the explanation for the ethnic disparities found in the literature.

### Arterial stiffness: pathophysiology

Studies have demonstrated the vast interference of cardiovascular risk factors on the pathophysiology of arterial stiffness. The mechanisms by which these risk factors lead to stiffening of the great arteries were progressively deciphered [15].

For instance, in patients with primary hypertension, arterial stiffness elevates as a result of the increase in vascular wall collagen, caused by high levels of blood pressure in repeated pulses, leading to biomechanical fatigue. Furthermore, activation of the renin-angiotensin-aldosterone system also influences this process by stimulating the proliferation of smooth muscle cells in the vessels, promoting low-grade inflammation, increasing collagen production, and favoring the formation of advanced glycation end products [16].

In individuals with type 2 diabetes, the damage to the walls of great arteries may derive from the main characteristics of the disease: hyperglycemia and insulin resistance [17]. Both factors act on structural and functional levels by various mechanisms. Chronic exposure to hyperglycemia leads to smooth muscle cells proliferation and increased production of advanced glycation end products, as well as to the promotion of collagen reticulation and stiffening of the arterial wall [33]. There is also an increase in the expression of matrix metalloproteinases 2 and 9 and angiotensin II receptors in the vascular tissue [34]. Insulin resistance increases collagen synthesis and the expression of many genes involved in inflammatory processes [35]. Arterial stiffness is a possible consequence of these phenomena.

In Chronic Kidney Disease (CKD), the process occurs essentially through vascular calcifications [36]. This is caused by a sequence of molecular events, beginning with the reduced expression of constitutive inhibitory proteins in smooth muscle cells, and increased expression of proteins related to the calcification process [37].

The Matrix Gla Protein (MGP) signaling pathway, in particular, contributes to the process of calcification and arterial stiffness. MGP is a small secretory protein produced by chondrocytes and vascular smooth muscle cells [38]. Its inactive form (desphospho-uncarboxylated MGP or dpucMGP) is carboxylated and phosphorylated to form active MGP, a potent arterial calcification inhibitor. This carboxylation depends on the disponibility of vitamin K on the vascular wall [38]. Curiously, vitamin K deficiency, even in low levels, seems to interfere **in** with this process. DpucMGP is secreted in the circulation, making it an interesting biomarker reflecting the vascular disponibility of vitamin K [38].

An increase in dpucMGP circulation is independently associated with an increase in arterial stiffness in many populations, including the general population [39,40] and adults with diabetes [41], hypertension [42], CKD [43], and Heart Failure (HF) [44]. Warfarin, a vitamin K antagonist, inhibits MGP activation and potently promotes vascular calcification in animal models. Its use has been associated with arterial stiffness in individuals with HF[44] and aortic valve calcification. There is data suggesting that vitamin K2 supplementation could reduce or improve arterial stiffness [45].

**Fig. 2:**
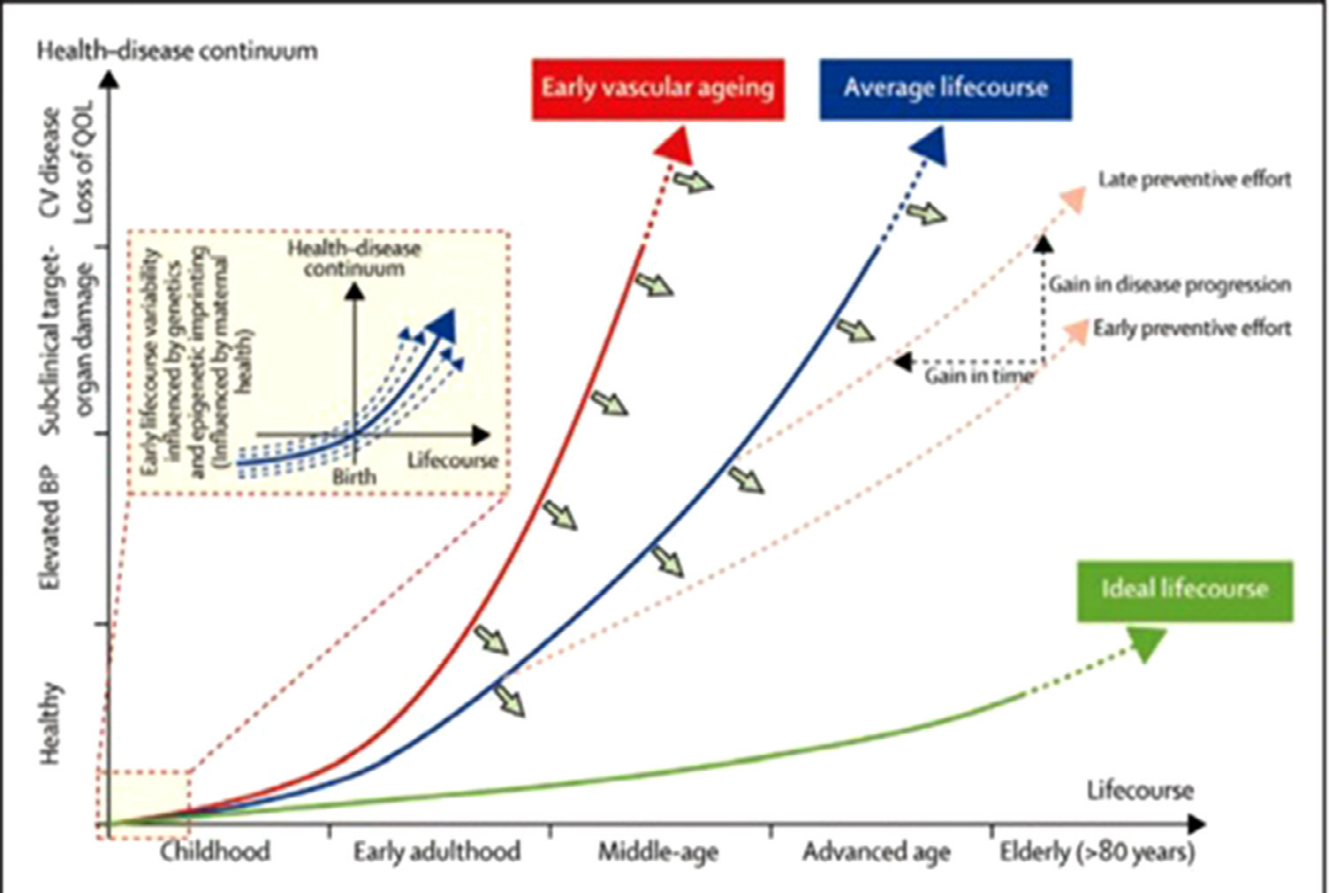
Adapted from Olsen et al [47].

This biological process does not affect every individual equally. Evidence in animal and human beings suggest that different segments of the aortic wall are affected heterogeneously [37,46]. Factors such as genetic susceptibility, epigenetic influence in intrauterine life, sociodemographic factors and health behaviors (including diet and exercise) all contribute to these differences, as illustrated in Fig. 2.

Increased arterial stiffness is frequently found in ethnic groups of lower socioeconomic status that have limited access to healthcare, inadequate diet, and unhealthy lifestyle [48], as stated in the previous section.

### Atrial consequences of arterial stiffness

Elevated arterial stiffness makes the transmitted pulse wave return from the terminal organs earlier, before the systole ends, increasing the pressure load over the left ventricle. Given the fact that different organs have different degrees of arterial pressure, consistent with the distance from the aorta and the anatomy of the vascular tree, registers of these waveforms vary according to the analyzed site [49], which explains the amplification of pulse pressure towards the peripheral vessels.

**Fig. 3:**
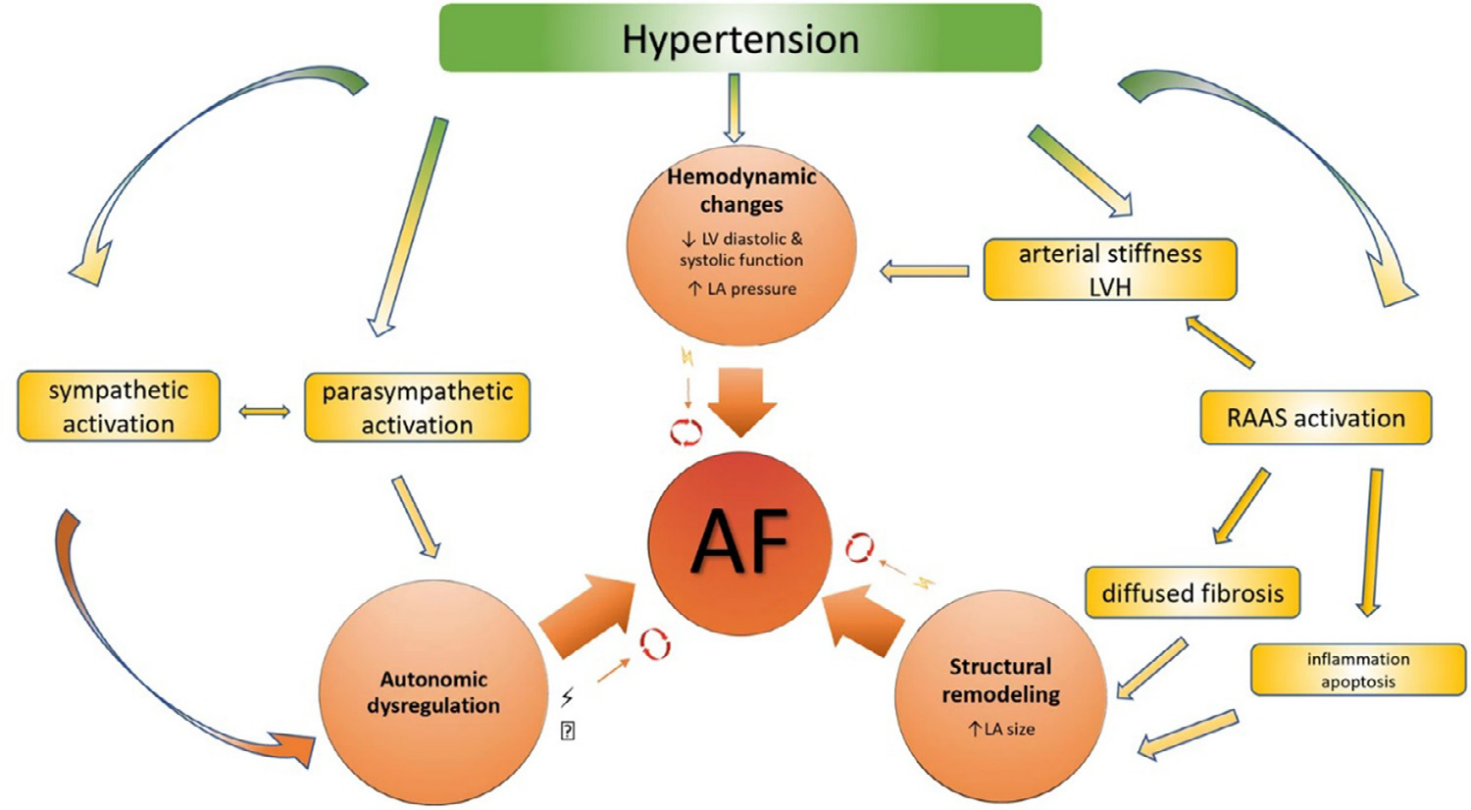
Adapted from Gumprecht et al [51].

A sub-analysis of the MESA Study demonstrated that Pulse Pressure (PP), but not median arterial pressure, was associated with a higher incidence of AF, even when adjusting for left atrium diameter and left ventricle mass index. An association between arterial stiffness and new AF episode has also been seen, even when adjusting for hypertension. Elevated “pulsatile load” ‒ that translates into elevated arterial stiffness ‒ would lead to left atrium distension and consequently neurohormonal activation leading to an inflammatory response, contributing to the development of AF [50], as illustrated in Fig. 3.

Chen et al. showed that patients with isolated AF (under 60 years of age and no history of diabetes, hypertension, coronary artery disease, HF, cardiomyopathy, hyperthyroidism or valvopathies), had a higher CFPWV, suggesting that early structural and functional changes in the arteries could be implicated in the pathogenesis of AF in these individuals [52].

In a population of 4264 Japanese individuals, Cui et al. identified an association between arterial stiffness (analyzed with the Augmentation Index) and new episodes of AF [53]. In the follow-up of a cohort of hypertensive patients in France, Cremer et al. demonstrated that arterial stiffness (analyzed by the QKD index) was associated with the occurrence of AF [54]. Also in France, Lantelme et al. showed PWV was associated with increased left atrium diameter and higher levels of NT-pro-BNP [55].

Yoshida et al. demonstrated for the first time, in a cohort of 1156 individuals, that arterial stiffness has a negative impact on left atrium function, assessed through transthoracic echocardiogram strain. The identified associations were independent of left ventricle volume and function, as well as BNP levels [56].

In echocardiography, left atrium peak systolic strain has been studied mainly in AF, where it was shown to be a factor for prognosis [57,58]. Shaik et al. found that it was significantly lower in AF patients when compared to healthy individuals [57]. Moreover, in AF patients submitted to electrical cardioversion, left atrium peak systolic strain was superior in patients that remained in sinus rhythm in comparison to those that had a recurrence of AF [57].

Additionally, a meta-analysis by Ma et al., with 686 total patients, showed that patients with recurrent AF after catheter ablation had higher values of left atrium peak systolic strain when compared to those that did not present recurrence. Finally, Parwani et al. demonstrated that reduced left atrium peak systolic strain (< 10%) was the most important factor associated with AF recurrence [59]. Therefore, it is possible to infer a strong association between atrial strain and atrial arrhythmia burden on follow-up.

More recently, in a Korean cross-sectional study with 8048 individuals, Chung et al. found that an increased arterial stiffness (assessed by the Cardiothoracic Vascular Index ‒ CAVI) is significantly associated with a higher prevalence of AF among those classified as being at intermediate and high risk by the Framingham Score [60].

Table 2 summarizes the main observational studies that studied the association between atrial fibrillation and arterial stiffness.

**Table 2:** Findings in previous observational studies.

Roetker et al [42].	
Prospective	Higher levels of systolic BP and PP, but not MAP or diastolic BP, were each individually associated with increased risk of AF after adjustment for all AF risk factors.
Chen et al.[44]	
Retrospective	After full adjustment, the odds ratios of AF were significantly higher per quartile increase in CFPWV and β index.
Cui et al [45].	
Cross-Sectional	The prevalence of atrial fibrillation and total arrhythmia were higher with larger AI (Augmentation Index) values.
Cremer et al [46].	
Prospective	Arterial stiffness (Assessed by QKDh) is a strong predictor of future atrial fibrillation in hypertensive patients, independently of age, 24-h pulse pressure and LAD.
Lantelme et al [47].	
Cross-Sectional	PWV was associated with increased left atrium diameter and higher levels of NT-pro-BNP.
Yoshida et al [48].	
Prospective	Arterial stiffness was independently associated with LA phasic function, even in the absence of overt cardiovascular disease, which may explain the higher incidence of atrial fibrillation in individuals with increased arterial stiffness.
Chung et al [68].	
Cross-Sectional	High arterial stiffness (assessed by CAVI) shows a significant association with AF in those with intermediate or high cardiovascular risk (Framingham) and can be used for further risk stratification of patients.

### Genetic aspects of arterial stiffness and atrial fibrillation

Regarding hypertensive individuals in general, a recent study by Hyman et al. demonstrated, through Mendelian randomization, that genetic variants related to increased arterial pressure were associated with a higher risk of AF, consistent with a causal relationship. These data support the concept that **to** lowering the blood pressure through pharmacological intervention could reduce the risk for AF. In the same study, interestingly, this last association was only significant for variants related to beta-blockers and calcium channel blockers target proteins [61].

The difference in effect size may also be due to the pleiotropic effects of these particular anti-hypertensive medications, as β-receptor signaling and calcium handling have both been implicated in AF initiation and arrhythmogenesis independent of blood pressure [61].

In another mendelian randomization study that assessed polymorphisms associated with increased arterial stiffness, it was shown that a genetic predisposition for a higher arterial stiffness index was unidirectionally associated with an increase in incidence risk and prevalence of AF. These results are consistent with a causality association between arterial stiffness and AF [62].

A similar study by Georgipoulos et al. presented the same causality relation between hypertension and atrial fibrillation, in which a 1 mm. Hg increase in systolic blood pressure increased the relative risk of AF development by 1.8%. For diastolic blood pressure and pulse pressure, this increase was 2.6% and 1.4%, respectively [63].

These data support the hypothesis of causality between arterial stiffness, hypertension, and atrial arrhythmias.

### Treatment and future perspectives

At this moment, there are no available drugs that directly promote arterial stiffness lowering. Nonetheless, most classes of anti-hypertensive drugs ‒ with most evidence for beta-blockers and diuretics, may be effective in reducing arterial stiffness. Among many patients with hypertension, an immediate decline in arterial stiffness may occur with anti-hypertensive therapy due to the reduction of the circumferential stress in the vessel.

In the long term, lower pressure levels allow for arterial wall remodeling, leading to lower stiffness. However, it cannot be assumed that anti-hypertensive therapy always improves arterial stiffness. The first randomized clinical trial to determine whether normalization of aortic stiffness improves cardiovascular outcomes, the SPARTE Trial, was recently published, showing no benefit in reducing cardiovascular events when guiding the treatment of hypertension by PWV when compared to the standard strategy by current guidelines, although there was a greater reduction in PWV in the group that guided the treatment by this parameter. It is important to emphasize that the work did not reach statistical power [64].

Many therapeutic strategies have been successful in animal models, including allopurinol and nitric oxide stimulants ‒ like nitrates, renin-angiotensin-aldosterone blockers ‒ such as angiotensin-converting enzyme inhibitors, angiotensin receptor blockers, and aldosterone receptor blockers (spironolactone). Although commonly prescribed in clinical practice for the treatment of hypertension and HF, they are not approved specifically for the reduction of arterial stiffness [65,66].

Exercise has also been demonstrated to have a positive modulator effect over arterial stiffness. A cross-sectional study showed an inverse relationship between exercise and stiffness, even with low-intensity physical activity. On the other hand, a sedentary lifestyle was associated with higher systolic blood pressure and PWV [65].

Other proposed therapies include the use of Dipeptidyl-Peptidase 4 (DPP4) inhibitors [66,67] and Sodium-Glucose Cotransporter 2 (SGLT2) inhibitors. One study found that switching from DPP4 inhibitors to tofogliflozin improved arterial stiffness in diabetic patients [68]. In another study with animal models, dapagliflozin improved vascular function in mice with type 2 diabetes [69].

A knowledge gap that is still to be clarified is whether a reduction in arterial stiffness leads to a decrease in the burden of atrial arrhythmias, from frequent atrial ectopies to AF. Another knowledge gap is whether there is an association between arterial stiffness and stages considered to be pre-fibrillatory that, once identified, would help in the understanding of this process.

## Conclusion

There is much evidence in the literature from different designs of observational studies indicating an association between arterial stiffness and atrial fibrillation. New studies exploring the association between arterial stiffness and pre-fibrillatory stages, and therefore, early stages of AF, would help corroborate the causality relationship between these two conditions.

Although much has been discovered since the first studies that related arterial stiffness to the progression of cardiovascular disease, the ability to clinically interrupt or revert the stiffness demands a much deeper understanding of the molecular, cellular and matrix interactions involved in this process.

## Authors’ contributions

Lage JGB - Conceptualization, - Data curation, - Formal analysis, Resources AND Writing - review & editing. Bortolotto LA - Conceptualization, - Data curation. Bortolotto AL - Data curation, Writing review and editing. Scanavacca MI - Supervision, - Validation, - Visualization Darrieux FCC - Supervision, - Validation, - Visualization Anyway.

## Declaration of Competing Interest

The authors declare no conflicts of interest.
